# Fundamental equations and hypotheses governing glomerular hemodynamics

**DOI:** 10.3389/fphys.2024.1440627

**Published:** 2024-08-14

**Authors:** Serena Y. Kuang, Besjana Ahmetaj, Xianggui Qu

**Affiliations:** ^1^ Department of Foundational Medical Studies, Oakland University William Beaumont School of Medicine, Rochester, MI, United States; ^2^ Department of Mathematics and Statistics, Oakland University, Rochester, MI, United States

**Keywords:** glomerular hemodynamics, renal plasma flow, glomerular filtration rate, efferent arteriole, mathematical model, colloid osmotic pressure, net filtration pressure, complex adaptive system

## Abstract

The glomerular filtration rate (GFR) is the outcome of glomerular hemodynamics, influenced by a series of parameters: renal plasma flow, resistances of afferent arterioles and efferent arterioles (EAs), hydrostatic pressures in the glomerular capillary and Bowman’s capsule, and plasma colloid osmotic pressure in the glomerular capillary. Although mathematical models have been proposed to predict the GFR at both the single-nephron level and the two-kidney system level using these parameters, mathematical equations governing glomerular filtration have not been well-established because of two major problems. First, the two-kidney system-level models are simply extended from the equations at the single-nephron level, which is inappropriate in epistemology and methodology. Second, the role of EAs in maintaining the normal GFR is underappreciated. In this article, these two problems are concretely elaborated, which collectively shows the need for a shift in epistemology toward a more holistic and evolving way of thinking, as reflected in the concept of the complex adaptive system (CAS). Then, we illustrate eight fundamental mathematical equations and four hypotheses governing glomerular hemodynamics at both the single-nephron and two-kidney levels as the theoretical foundation of glomerular hemodynamics. This illustration takes two steps. The first step is to modify the existing equations in the literature and establish a new equation within the conventional paradigm of epistemology. The second step is to formulate four hypotheses through logical reasoning from the perspective of the CAS (beyond the conventional paradigm). Finally, we apply the new equation and hypotheses to comprehensively analyze glomerular hemodynamics under different conditions and predict the GFR. By doing so, some concrete issues are eliminated. Unresolved issues are discussed from the perspective of the CAS and a desinger’s view. In summary, this article advances the theoretical study of glomerular dynamics by 1) clarifying the necessity of shifting to the CAS paradigm; 2) adding new knowledge/insights into the significant role of EAs in maintaining the normal GFR; 3) bridging the significant gap between research findings and physiology education; and 4) establishing a new and advanced foundation for physiology education.

## 1 Introduction

The mammalian kidney is a vital organ responsible for maintaining homeostasis by regulating fluid balance, electrolytes, and waste removal through urine production. It plays a crucial role in the overall health and functionality of the body. The kidney is unique in the body as it is the only organ that has two arterioles and two capillary beds aligned in a series. The renal autoregulation mechanisms (myogenic response and tubuloglomerular feedback [TGF]) keep the renal plasma flow (RPF) and glomerular filtration rate (GFR) stable when arterial blood pressure fluctuates within a broad range. This indicates the importance of maintaining a normal GFR that is critical to the homeostasis of the internal environment. Precise regulation of the GFR depends on the balance between the resistances of afferent arterioles (AAs) and efferent arterioles (EAs), which together determine the net filtration pressure (NetP) that drives glomerular filtration.

NetP is the sum of the four Starling forces across a glomerular capillary wall. The two Starling forces that favor glomerular filtration are the hydrostatic pressure in the glomerular capillary (P_GC_) and the colloid osmotic pressure in Bowman’s capsule (π_BC_), where π_BC_ is zero or negligible in the normal situation and becomes significant in patients with various renal diseases. The two Starling forces that oppose glomerular filtration are the plasma colloid osmotic pressure in the glomerular capillary (π_GC_) and the hydrostatic pressure in Bowman’s capsule (P_BC_). The filtration fraction (FF) is the ratio of the GFR/RPF and is about 20% in the normal situation. All of these parameters together characterize glomerular hemodynamics and determine the GFR.

The parameters of glomerular hemodynamics are well-known and have been used to establish mathematical models to predict the GFR at both the single-nephron (SN) level and the two-kidney system level (Deen et al., 1974; Huss et al., 1975; Brenner et al., 1976; Navar et al., 1977; Chang, 1978; Papenfuss and Gross, 1978; Tucker and Blantz, 1981; Sgouralis and Layton, 2015). Nevertheless, two major problems exist: first, the two-kidney system-level equations are simply extended from the equations at the SN level, which is inappropriate in epistemology and methodology. Second, EAs play an important role in glomerular hemodynamics and, thus, the GFR, but the role of EAs in the maintenance of the normal GFR is underappreciated. These two problems are elaborated in terms of a total of six concrete issues in the next section and collectively show the need for a shift in epistemology toward a more holistic and evolved way of thinking reflected in the concept of the complex adaptive system (CAS; Holland, 2006; Carmichael and Hadžikadić, 2019).

After this elaboration, we illustrate eight fundamental equations and four hypotheses that govern glomerular hemodynamics at both the SN and two-kidney system levels as the theoretical foundation of glomerular hemodynamics. This illustration modifies some equations in the literature, establishes a new equation in the conventional paradigm of epistemology, and formulates four new hypotheses through logical reasoning from the perspective of the CAS (beyond the conventional paradigm). Finally, we apply the new equation and hypotheses we established to comprehensively analyze glomerular hemodynamics under different conditions and predict the GFR. By doing so, some concrete issues are eliminated. Unresolved issues are discussed from the perspective of the CAS and a desinger’s view. The methodology in this article is logical, largely quantitative, and systematic.

The significance of this article is as follows: 1) it makes clear the necessity of shifting the epistemology that guides research from a conventional paradigm toward a CAS paradigm; 2) it adds new knowledge/insights to understand the significant potential role of EAs in maintaining the normal GFR, which has been underappreciated; 3) it bridges the significant gap between research findings and physiology education; and 4) it establishes a new and advanced foundation for physiology education in which glomerular hemodynamics should be illustrated at the SN and two-kidney system levels.

## 2 How are system-level equations extended from the SN level, and why is the role of EAs in the GFR underappreciated?

**FIGURE 1:**
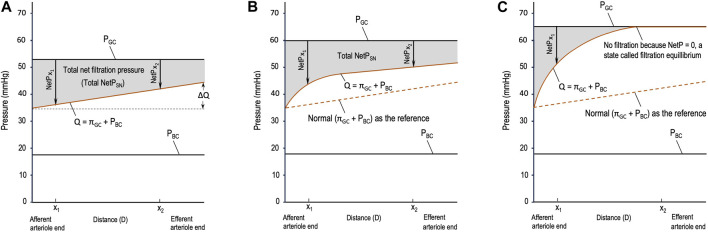
Starling forces across the wall of a glomerular capillary (GC) in three different situations. **(A)** Normal stable glomerular hydrostatic pressure (P_GC_) and increasing plasma colloid osmotic pressure (π_GC_); **(B)** initial moderate elevations in P_GC_ and π_GC_ due to moderate efferent arteriole (EA) constriction; and **(C)** initial significant elevations in P_GC_ and π_GC_ due to severe EA constriction. P_BC_: hydrostatic pressure in Bowman’s capsule, x_1_ and x_2_: distances in the capillary from the afferent arteriole end.

Different from the pressure profile in the peripheral capillaries, the decrease in P_GC_ during glomerular filtration is insignificant, so P_GC_ is constant throughout glomerular filtration (Figure 1). Meanwhile, π_GC_ is a variable that increases linearly (Figure 1A) or nonlinearly (Figures 1B,C) during glomerular filtration (Brenner et al., 1976; Giebisch et al., 2017; Hall and Hall, 2021). The rising orange line labeled Q in Figure 1 shows the sum of the two opposing pressures to glomerular filtration: π_GC_ + P_BC_. P_BC_ is constant under normal conditions (Giebisch et al., 2017) and in Figure 1, and π_BC_ is ignored under normal situations and, thus, not drawn. If the orange line rises, it means that π_GC_ increases because P_BC_ remains constant.

Based on Figure 1, multiple issues can be addressed as follows. In the literature, different symbols are used to refer to the parameters of glomerular hemodynamics. In this article, we deal with the equations that model glomerular hemodynamics at both the SN and system levels; hence, the parameters at these levels are clearly differentiated to avoid confusion, and new terms are defined when necessary.

### 2.1 Issue 1

The widely used equation, NetP = P_GC_ – π_GC_ – P_BC_, is qualitative and vague. π_GC_ increases during glomerular filtration, so it is unclear which value of π_GC_, such as an instantaneous π_GC_ or the mean π_GC_ throughout glomerular filtration ( $\bar{\pi_{\text{GC}\_\text{SN}}}$ ), should be used in the equation. It is also unclear how $\bar{\pi_{\text{GC}\_\text{SN}}}$ should be quantified when it increases nonlinearly (Figures 1B,C). Subsequently, whether NetP is instantaneous or the total NetP_SN_ or the mean NetP throughout glomerular filtration ( $\bar{{\text{NetP}}_{\text{SN}}}$ ) is not addressed. These questions are sometimes addressed in research (Brenner et al., 1976; Navar et al., 1977; Papenfuss and Gross, 1978) but do not appear in physiology education, leaving the equation quantitatively inappropriate and useless.

### 2.2 Issue 2

Another widely used equation, GFR = K_f_(NetP) = K_f_(P_GC_ – π_GC_ – P_BC_), where K_f_ refers to the filtration coefficient (Drumond and Deen, 1994; Leatherby et al., 2021), is more vague or ambiguous. It has the same problem addressed above. Moreover,

• P_GC_, π_GC_, and P_BC_ are pressures across a glomerular capillary wall, whereas the GFR is the filtration achieved by the two kidneys (with numerous nephrons) per unit time. It is inappropriate to calculate the GFR at the system level using these single-capillary-level pressures.
• The filtration coefficient is the product of the hydraulic permeability of the filtration membrane (K or L_P_A) and the filtration area (Brenner et al., 1976; Tucker and Blantz, 1977; Hall and Hall, 2021). The total filtration area of the two kidneys is remarkably different from the filtration area of an SN. However, the symbols that refer to them are inconsistent: either SNK_f_ or K_f_ is used to refer to the filtration coefficient at the SN level (Brenner et al., 1976; Ott et al., 1976; Navar et al., 1977; Marchand and Mohrman, 1980; Savin and Terreros, 1981; Arendshorst and Gottschalk, 1985), whereas K_f_ also refers to the filtration coefficient at the two-kidney level (Giebisch et al., 2017; Hall and Hall, 2021). It should be noted that the unit of the filtration coefficient for the SN is nl/min/mmHg (Brenner et al., 1976; Navar et al., 1977; Marchand and Mohrman, 1980; Savin and Terreros, 1981) or nl/sec/mmHg (Tucker and Blantz, 1977; Chang, 1978; Arendshorst and Gottschalk, 1985), whereas the unit of the coefficient for the two-kidney system is ml/min/mmHg (Costanzo, 2018; Hall and Hall, 2021).
• It is unclear what NetP refers to in the equation. In other words, it remains unclear whether it should be $\bar{{\text{NetP}}_{\text{SN}}}$ averaged at the SN level or the mean NetP averaged from numerous nephrons at the system level ( $\bar{{\text{NetP}}_{\text{SYS}}}$ ).
• If NetP in this equation applies to an SN, then it should be total NetP_SN_ or $\bar{{\text{NetP}}_{\text{SN}}}$ , the GFR should be SNGFR, and the filtration coefficient should be SNK_f_. If NetP refers to $\bar{{\text{NetP}}_{\text{SYS}}}$ , then the GFR should remain the GFR; the filtration coefficient should be K_f_; and P_GC_, π_GC_, and P_BC_ should be averaged at the system levels $\bar{P_{\text{GC}\_\text{SYS}}}$ , $\bar{\pi_{\text{GC}\_\text{SYS}}}$ , and $\bar{P_{\text{BC}\_\text{SYS}}}$ , respectively.

To the best of our knowledge, this is the first time that two sets of parameters have been defined and differentiated systematically to avoid confusion. Specifically, P_GC_, P_BC_, dπ_GC_/dx, $\bar{\pi_{\text{GC}\_\text{SN}}}$ , SNK_f_, d(NetP)/dx, total NetP_SN_, SNRPF, SNGFR, and SNFF apply to the SN level, and RPF, GFR, FF, K_f_, $\bar{{\text{NetP}}_{\text{SYS}}}$ , $\bar{P_{\text{GC}\_\text{SYS}}}$ , $\bar{\pi_{\text{GC}\_\text{SYS}}}$ , and $\bar{P_{\text{BC}\_\text{SYS}}}$ apply to the system level.

### 2.3 Issue 3

Whether filtration equilibrium occurs in mammalian kidneys remains a continuous debate (Osgood et al., 1982; Arendshorst and Gottschalk, 1985). Filtration equilibrium refers to the phenomenon when NetP decreases to zero at a point before the blood reaches the EA so that no filtration occurs after this point (Figure 1C). Filtration equilibrium has been reported in some experimental studies on Munich–Wistar rats (Marchand and Mohrman, 1980) and squirrel monkeys (Maddox et al., 1974) but has not been observed in studies on dogs (Ott et al., 1976), Wistar rats (Seiller and Gertz, 1977), and rabbits (Denton and Anderson, 1991). In other words, glomerular filtration in the kidneys of these animals is characterized by filtration disequilibrium.

### 2.4 Issue 4

A critical gap in current research is the notable lack of research questions and efforts to investigate whether there is a direct and/or indirect communication between an upstream AA and its downstream EA. This situation may lead to missing crucial insights into glomerular hemodynamics. The advantages of these types of communication are obvious. For example, in the design of artificial nephrons or kidneys, enabling these communications could potentially improve the coordination between AAs and EAs and lead to more efficient function. On the other hand, Davis (1991) reported that under certain circumstances, TGF may involve EA vasomotion either in the same or opposite direction of AA vasomotion. If there is no communication between EAs and AAs, necessary vasomotion of AAs and/or EAs to maintain the normal GFR may be mediated through the TGF. If so, it is neither efficient nor economical.

### 2.5 Issue 5

Physiologists often note that FF = GFR/RPF ≈ 20%. Obviously, this means that a much larger fraction of RPF (80%) is not filtered but exits through the EAs under normal conditions. This 80% fraction is apparently ignored because its implications for glomerular hemodynamics and maintaining the normal GFR are not mentioned, appreciated, or discussed in the literature. The importance of having 80% RPF exiting through EAs becomes clear gradually in this article, and its implications are addressed in *Conclusion*.

### 2.6 Issue 6

In terms of how AA resistance influences glomerular hemodynamics, there is no disagreement among physiologists in general. However, the explanations of how EA resistance influences glomerular hemodynamics are inconsistent, incomplete, and inappropriate:

• Some literature only introduce the effect of AA resistance on the GFR but not the effect of EA constriction (Pal et al., 2017; Kibble, 2020; Loscalzo et al., 2022; Eaton and Pooler, 2023).
• EA constriction may increase both P_GC_ and π_GC_ but may or may not reduce RPF. However, textbooks often merely mention that EA constriction increases P_GC_ and/or GFR and do not address how it influences RPF and/or π_GC_ (Bijlani and Manjunatha, 2011; Krishna, 2015; Koeppen and Stanton, 2018; Barrett et al., 2019).
• Some literature briefly note that EA constriction has a biphasic effect on the GFR depending on whether EA constriction reduces RPF and how significantly it increases π_GC._ Specifically, if the EAs constrict slightly, which reduces RPF insignificantly or not at all, then the GFR increases slightly. However, if the EAs constrict severely (causing a threefold or more increase in the EA resistance), the RPF and GFR are both reduced because under this circumstance, the increase in π_GC_ (∆π_GC_) becomes greater than the increase in P_GC_ (∆P_GC_), i.e., ∆π_GC_ > ∆P_GC_ (Giebisch et al., 2017; Hall and Hall, 2021). This means that the role of each phase of the biphasic effect of EA constriction on the RPF and, thus, GFR is conditional.

Omitting the analysis of RPF, π_GC_, and whether ∆π_GC_ > ∆P_GC_ but stating that EA constriction increases or decreases the GFR, is logically flawed. The comparison between ∆π_GC_ and ∆P_GC_ is an indispensable step that determines whether the GFR increases or remains unchanged or decreases in response to a change in EA resistance. However, comparing the two is not appropriate because of the lack of logical rigor, as shown in issue 1 above. Theoretically, ∆π_GC_ > ∆P_GC_ needs to be replaced by $\bar{{∆\pi }_{\text{GC}\_\text{SN}}}$ > ∆P_GC_ or a comparison of the total NetP_SN_ in a situation with the normal total NetP_SN_ illustrated in Section 4 (see Hypothesis 1).

### 2.7 Summary

These issues, together, indicate the following:

• The qualitative, vague equations cause insufficient and confusing definitions of the parameters at both the SN and system levels.
• A system-level understanding of glomerular hemodynamics is mechanically extended from the SN level due to the lack of an appropriate epistemology.
• A comprehensive understanding of the role of the EAs as a type of resistance vessel on glomerular hemodynamics and, thus, the GFR has not been well-established.

## 3 Eight fundamental mathematical equations within the conventional paradigm of epistemology

The mathematical equations illustrated in this section can be reasoned out by anyone who understands the fundamentals of calculus or can be modified from the literature Eq. 1, Eq. 2, Eq. 6, and Eq. 7. SN-level parameters (SNRPF, d(NetP)/dx, total NetP_SN_, $\bar{{\text{NetP}}_{\text{SN}}}$ , $\bar{\pi_{\text{GC}\_\text{SN}}}$ , SNK_f_, SNGFR, and SNFF) and system-level parameters (RPF, $\bar{{\text{NetP}}_{\text{SYS}}}$ , $\bar{P_{\text{GC}\_\text{SYS}}}$ , $\bar{\pi_{\text{GC}\_\text{SYS}}}$ , $\bar{P_{\text{BC}\_\text{SYS}}}$ , K_f_, GFR, and FF) are easy to differentiate. Following common practice in the literature, all capillaries in a glomerulus are considered one tube with the same filtration area as all the capillaries together (Brenner et al., 1976; Chang, 1978; Drumond and Deen, 1994).

Since π_GC_ is the function of the distance (x) from a point of glomerular filtration to the beginning of the filtration (the AA end of the capillary), the vague expression NetP = P_GC_ – π_GC_ – P_BC_ needs to be derived to quantify a derivative NetP [d(NetP)/dx)] and the total NetP_SN_ [the integration of d(NetP)/dx)]:

$$d\left(\text{NetP}\right)/\text{dx}=P_{\text{GC}}\;–\;\pi_{\text{GC}}\left(x\right)\;‐\;P_{\text{BC}}$$ (1)

and

$$\text{Total }\;{\text{NetP}}_{\text{SN}}=\int_{\text{AA}\;\text{end}}^{\text{EA}\;\text{end}}\left[P_{\text{GC}}-\;\pi_{\text{GC}}\left(x\right)\;-\;P_{\text{BC}}\right]\text{dx}=\int_{\text{AA}\;\text{end}}^{\text{EA}\;\text{end}}\left(P_{\text{GC}}-\text{ mQ}\right)\text{dx },$$ (2)

where Q = $\pi_{\text{GC}}+P_{\text{BC}}$ , m = $\frac{∆Q}{D}$ represents the slope of the orange line in Figure 1A, and D refers to the distance from the AA end to the end of glomerular filtration. The gray area in Figure 1A represents the total NetP_SN_ determined by Eq. 2.

If the orange line is a curve (Figures 1B,C), integrating the total NetP_SN_ becomes complex. It requires conducting experiments, setting points to collect data, and then performing mathematical modeling, which is beyond the scope of these fundamental equations. Nevertheless, regardless of whether the total NetP_SN_ can be easily integrated using Eq. 2 or needs a complex model, the gray area in Figures 1A–C represents the total NetP_SN_ determined by the line of P_GC_ and the orange line.

Furthermore, an instantaneous SNGFR can be reasoned out or modified from the equation provided by Brenner et al. (1976) or Navar et al. (1977) using the symbols at the SN level defined in this article:

$$D\left(\text{SNGFR}\right)/\text{dx}={\text{SNK}}_{f}\left[\text{d}\left(\mathrm{NetP}\right)/\;\text{dx}\right]={\text{SNK}}_{f}\left[P_{\text{GC}}\;–\;\pi_{\text{GC}}\left(x\right)\;-\;P_{\text{BC}}\right].$$ (3)

The total SNGFR can be reasoned out as follows or modified from the equation provided by Deen et al. (1974) or Sgouralis and Layton (2015) using the symbols at the SN level defined in this article:

$$\text{Total SNGFR}={\text{SNK}}_{f}\;\int_{\text{AA}\;\text{end}}^{\text{EA}\;\text{end}}\left[P_{\text{GC}}\;–\;\pi_{\text{GC}}\left(x\right)\;‐\;P_{\text{BC}}\right]\text{dx}.$$ (4)

Similarly, the vague expression GFR = K_f_(NetP) = K_f_(P_GC_ – π_GC_ – P_BC_) needs to be derived to estimate the GFR using the symbols at the two-kidney system level defined in this article:

$$\text{GFR}=\left(K_{f}\right)\bar{{\text{NetP}}_{\text{SYS}}}=K_{f}\bar{P_{\text{GC}\_\text{SYS}}\;}\;–\;\bar{\pi_{\text{GC}\_\text{SYS}}}\;–\;\bar{P_{\text{BC}\_\text{SYS}}}).$$ (5)

Obviously, the many mean values in the equation can only be estimated for the millions of nephrons at the two-kidney level. This equation makes better sense than its original form [GFR = K_f_(NetP) = K_f_(P_GC_ – π_GC_ – P_BC_)]. It is theoretically meaningful but is still of no practical use. Practically and clinically, the GFR can be calculated using inulin clearance or estimated using creatinine clearance.

The following equation estimates K_f_:

$${\text{K}}_{\text{f}}={\text{SNK}}_{\text{f}}\;\text{x}\;\text{estimated}\;\text{total}\;\text{number}\;\text{of}\;\text{nephrons}\;\text{in}\;\text{the}\;\text{two}\;\text{kidneys}$$ (6)

Hladunewich et al. (2004) estimated K_f_ for pregnant women using this equation, where the estimated mean total number of nephrons for healthy women between the ages of 20 and 50 years is 1.4 × 10^6^ (Nyengaard and Bendtsen, 1992).

The relationship between the GFR and SNGFR can be expressed as

$$\text{GFR}=\sum_{1}^{\text{Total}\;\text{number}\;\text{of}\;\text{glomeruli}\;\text{in}\;\text{two}\;\text{kidneys}}\text{SNGFR}.$$ (7)

Since RPF is the plasma flow that enters AAs and FF = GRF/RPF ≈ 20%, we establish the following equation to describe the distribution of RPF after entering AAs at the two-kidney system level:

$$\text{RPF}=\text{GFR}+{\text{RPF}}_{\text{EA}}\approx 20\%\text{ RPF}+80\%\text{ RPF}.$$ (8)

Eq. 8 leads to the formulation of the last hypothesis in the next section and is critical to resolve issue 6 and understand the significant potential role of EAs in maintaining the normal GFR when renal autoregulation fails to maintain the normal RPF.

The two kidneys as a whole have numerous nephrons (about 30,000 in a rat kidney and 10^6^ in a human kidney; Sgouralis and Layton, 2015). These nephrons not only have similarities in their structures and functions but also exhibit heterogeneity in their structural and functional aspects from the molecular level to the cellular, nephron, and regional levels. All of these contribute to the complexity of the system (the two kidneys).

Moreover, a system with numerous components exhibits emergent properties that its components or agents (in this context, single nephrons) do not possess, such as a great capacity of resilience and adaptability to internal and external perturbations, as well as nonlinearity. Nonlinearity means that the response of such a system toward a perturbation is often unproportional to the strength of the perturbation (Janson, 2012), and a perturbation to the system may cause a large nonproportional response, a proportional response, or no response at all. For instance, Denton et al. (2000) reported that administering intrarenal angiotensin II caused a decrease in RPF with a concomitant increase in FF in a dose-dependent manner so that the GFR does not decrease but is maintained with no change. Their research also showed the following observations:

• From the outer cortex to the juxtamedullary cortex, the diameters of the EAs show a gradient: those with the smallest diameters are in the outer cortex, whereas those with the largest diameters lie in the juxtamedullary cortex.
• The diameters of the EA in the outer and mid cortexes are smaller than those of the AA, but the diameters of the EA in the juxtamedullary cortex are similar to those of the AA.
• Such heterogeneity in the diameters of the EA seems to be one of the reasons that angiotensin II has differential degrees of vasoconstrictive effects on the AAs and EAs. According to Poiseuille’s equation, which states that resistance is inversely proportional to the fourth power of the radius, it can be predicted that the EAs in the outer cortex with the smallest diameters can impact glomerular hemodynamics most substantially.
• Since outer and midcortical glomeruli account for about 90% of all glomeruli, in general, angiotensin II tends to cause a higher increase in EA resistance than in AA resistance.

Therefore, the sensitivity of glomerular hemodynamics to a minor change in EA resistance should not be ignored. In other words, the EAs possess great potential to regulate glomerular hemodynamics in various ways to maintain a normal GFR. If we consider the two kidneys a renal CAS (Holland, 2006; Carmichael and Hadžikadić, 2019) with both similarities and heterogeneities in its agents and emergent properties at the system level, it becomes clear why extending the SN-level equations to describe the system-level equations is inappropriate in epistemology and methodology. However, so far, mathematical modeling of a CAS is difficult and needs further advancement.

From the perspective of the CAS and a designer’s view, the debate about whether filtration equilibrium does or does not occur in mammalian nephrons may be reconsidered to avoid a mechanical, mutually exclusive approach and facilitate a holistic and dynamic approach:

• “Fitness functions that are inherent in nature are always pushing the system, any system, toward more efficient use of resources” (Carmichael and Hadžikadić, 2019). Filtration equilibrium makes a fraction of the capillary useless for filtration. Therefore, it is worth considering that filtration equilibrium might not be the normal condition and may occur only under specific circumstances. Theoretically, if SNRPF is low and/or SNK_f_ is high and/or the constriction of an EA is severe, filtration equilibrium may occur (Arendshorst and Gottschalk, 1985) in some glomeruli. Practically, multiple factors may encourage or prevent it. It is crucial to identify these factors and determine whether filtration equilibrium is more or less likely to occur in specific regions of the kidney.
• If we design an artificial kidney, it is important to determine whether it is beneficial for filtration equilibrium and disequilibrium to be mutually transformable under some conditions for the sole purpose of increasing the capacity of both resilience and adaptability of the kidneys. Alternatively, it should be assessed whether filtration equilibrium should be more likely to appear in some nephrons and less likely to occur in others for the same purpose.
• Renal heterogeneity could be a consequence of the past adaptive processes of the renal CAS toward internal and external perturbations for the purpose of maintaining a normal GFR. It is essential to explore whether renal heterogeneity should exhibit different patterns at various levels, from molecular to cellular, nephron, and system, in response to different perturbations.
• Developing methods to study and recognize different patterns of renal heterogeneity is critical for advancing our understanding of kidney function.

The eight equations given above are generally linear or simple models; thus, we consider them fundamental in the study of glomerular hemodynamics, or more specifically, glomerular filtration. To model other aspects of glomerular hemodynamics or, more broadly, renal hemodynamics, such as renal autoregulation (myogenic response and TGF) and coupled nephrons, much more complex mathematical models are needed, and readers may refer to the review article by Sgouralis and Layton (2015). Like the eight fundamental mathematical models mentioned above, complex models have the same problem, i.e., how the SN and two-kidney levels of models can be well-integrated by taking both the similarity and heterogeneity of nephrons into consideration in the direction of the CAS.

## 4 Four hypotheses from the perspective of the complex adaptive system

Obviously, the gray area in Figure 1C due to severe EA constriction is smaller than the normal gray area in Figure 1A. If the SNGFR were to be calculated for the condition in Figure 1C, it would be less than the normal SNGFR in Figure 1A. Depending on the concrete value of SNRPF, the exact degree of EA constriction, and the resulting P_GC_ and π_GC_, the gray area due to moderate EA constriction (Figure 1B) may be greater than, equal to, or smaller than the normal gray area in Figure 1A. Note that in Figure 1B, before x_1_, NetP (x < x_1_) is greater than that in the normal situation (Figure 1A); after x_1_, NetP (x > x_1_) becomes smaller than normal; and at x_1_, NetP (x_1_, Figure 1B) = the normal (x_1_ in Figure 1A)^1^ (this analysis also applies to Figure 1C). The closer x_1_ is to the AA end in Figure 1B, the more likely it is that the resulting gray area is smaller than the normal gray area in Figure 1A; on the contrary, the farther x_1_ is from the AA end in Figure 1B, the more likely the gray area is to be equal to or greater than the normal gray area in Figure 1A. This analysis makes it clearer that a comparison of whether ∆π_GC_ > ∆P_GC_ is not practical, but a comparison of a gray area with the normal gray area is doable. Hence, logically, the comparison of ∆π_GC_ and ∆P_GC_ should be replaced by Hypothesis 1:

Hypothesis 1 If the total NetP_NS_ (the gray area) < or = or > the normal, then SNGFR < or = or > the normal. In other words, the SNGFR decreases or remains unchanged or increases.

Hypothesis 1 is qualitative and specifically useful for physiology education, which is so far not math-heavy. From now on, if the total NetP_SN_ is reduced compared to the normal, it means that the mean increase in π_GC_ is greater than the increase in P_GC,_ i.e., $\bar{{∆\pi }_{\text{GC}\_\text{SN}}}$ > ∆P_GC_. Subsequently,

Hypothesis 2 If the total NetP_SN_ in a significant number of nephrons increases/decreases, $\bar{{\text{NetP}}_{\text{sys}}}$ increases/decreases so that the GFR increases/decreases.

Note that from the total NetP_SN_ at the SN level to $\bar{{\text{NetP}}_{\text{sys}}}$ at the system level, the description “significant number of nephrons increases/decreases” reflects the CAS. Since renal autoregulation mechanisms conventionally involve the AA and macula densa, not the EA, from a designer’s view, we hypothesize that there are unknown direct or indirect communications (mechanical, electrical, chemical, or biological) between an upstream AA and its downstream EA, or between a glomerulus and its downstream EA, or between the macula densa and its adjacent EA, so that these parties work efficiently and in coordination to precisely maintain the normal GFR as far as possible, especially when renal autoregulation mechanisms fail to maintain stable RPF:

Hypothesis 3 If the GFR is greater than the normal upper range, *generally speaking*, the AAs should constrict, or the EAs should dilate, or both; on the contrary, if the GFR is less than the normal lower range, the AAs should dilate or the EAs should constrict or both.

To date, the occasional involvement of the EA in TGF has been reported under some circumstances (Davis, 1991; Ren et al., 2001); some autocoids (e.g., nitric oxide and prostgalndins etc.) produced in the glomerulus may diffuse to influence EA vasomotion (Ito and Abe, 1997; Leipziger and Praetorius, 2020). Chilton et al. (2008) speculated a direct communication between an upstream AA and its downstream EA via electrical potential change in the smooth muscles of the EA. Denton et al. (2000) reported that angiotensin II changed the geometry of the glomerular pole including the extraglomerular mesangium, whereas Elger et al. (1998) suggested a direct functional influence of an AA on an EA via the extraglomerular mesangium and the presence of a specific sheer stress receptor located in the intraglomerular portion of the EA. Further research to explore the hypothesized communications between an upstream AA and its downstream EA will be of great value. In Section 5, we show the pivotal role of this hypothesis in guiding our analyses of glomerular hemodynamics and GFR under various conditions and eventually resolve issue 6.

Next, we reason out Hypothesis 4 using the following data. If the RPF of a healthy man is approximately 600 mL/min, the GFR is approximately 125 mL/min, and FF is approximately 20%, then his RPF_EA_ (renal plasma flow that exits through the EAs) should be approximately 475 mL/min. Below, the unit ml/min is omitted for RPF, GFR, and RPF_EA_. Assume that his blood pressure decreases too much for some reason so that renal autoreglation can no longer maintain stable RPF, e.g., RPF decreases to 300. In order to maintain the GFR at approximately 125, according to Eq. 8, the EAs should constrict to cause RPF_EA_ to be approximately 175. If RPF_EA_ > or <175, then GFR < or > normal, indicating that the total NetP_SN_ in a significant number of nephrons in the two kidneys < or > their normal values. Hence, 175 is the critical point of RPF_EA_ in response to the primary change of RPF = 300. If RPF is approximately 400, then the critical point of RPF_EA_ should be approximately 275. Due to the heterogeneity of the nephrons, it is not easy to obtain or estimate the total NetP_SN_ for the majority of the nephrons. The purpose of introducing the concept of the critical point of RPF_EA_ at the two-kidney level is to use it to estimate what is more likely to happen in the majority of the nephrons in terms of their total NetP_SN_ when RPF is below normal:

Hypothesis 4a If RPF_EA_ > or = or < a critical point in response to a particular value of RPF, the total NetP_SN_ in a significant number of nephrons is < or = or > their normal values.

On the contrary, if EA vasomotion is the primary change, then RPF has a critical point in response to a particular EA resistance, which also predicts the total NetP_SN_:

Hypothesis 4b If RPF > or = or < a critical point in response to a particular value of RPF_EA_, the total NetP_SN_ in a significant number of nephrons is > or = or < their normal value.

Hypothesis 4 is inferred from Eq. 8. This is the first significance of Eq. 8.

The illustration of the equations and hypotheses leads to the criteria to define the following terms in this article:

• Slight EA constriction means that the EAs constrict slightly, which does not reduce RPF but redistributes it to the GFR and RPF_EA_.
• Severe EA constriction means that the EAs constrict significantly, causing a threefold or more increase in EA resistance and reducing RPF, which results in the total NetP_SN_ in the majority of the nephrons becoming smaller than normal (Figure 1C) and, thus, a decrease in $\bar{{\text{NetP}}_{\text{sys}}}$ and the GFR.
• Moderate EA constriction is between slight and severe constriction, which may lead to an increase or decrease or no change in the GFR depending on the resulting RPF, P_GC_, π_GC_, and total NetP_SN_ in the majority of the nephrons, as shown in Figure 1B.

**FIGURE 2:**
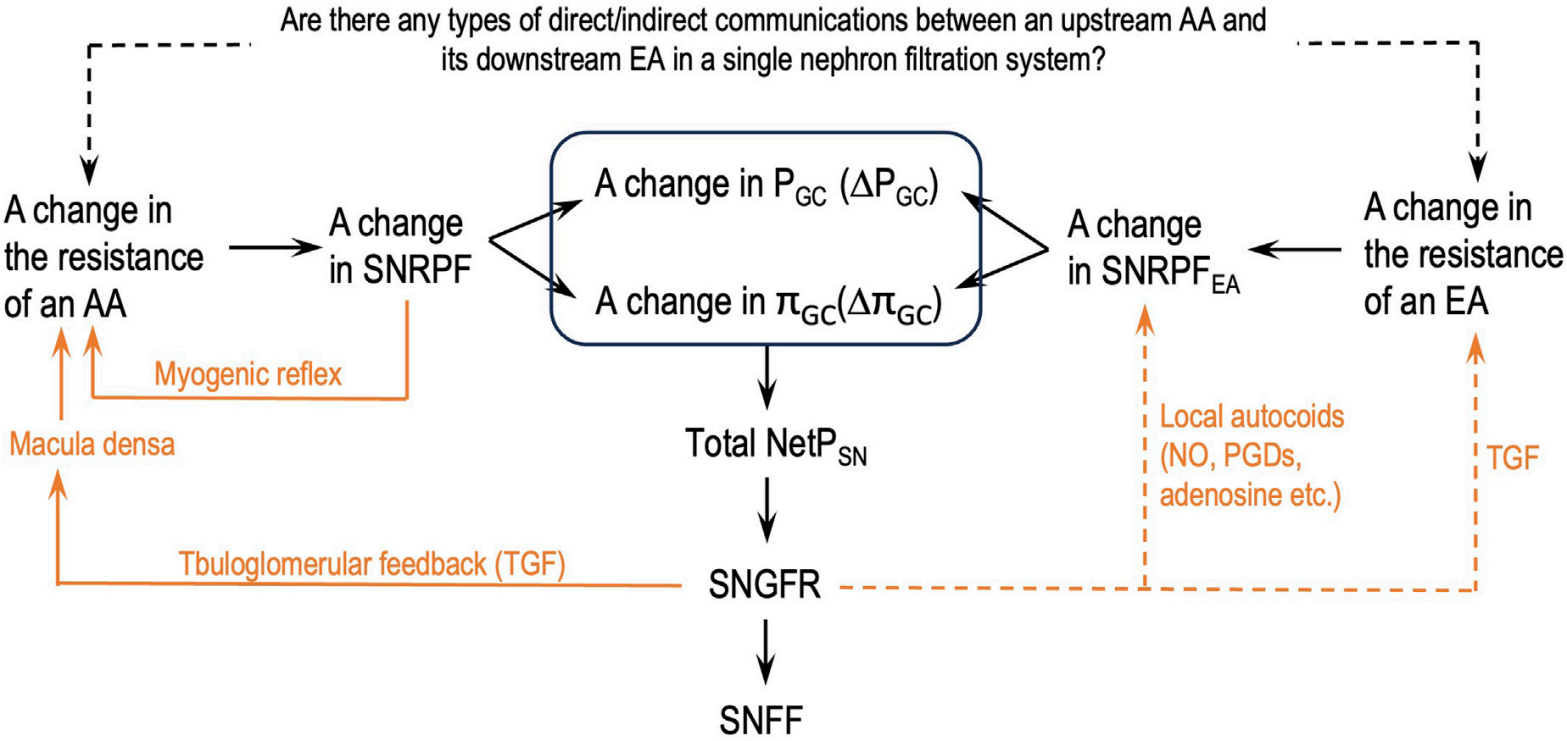
Logical steps to analyze the parameters of the glomerular hemodynamics of a single nephron after a change in the resistance of either an afferent arteriole (AA) or an efferent arteriole (EA) or both. SNGFR: single-nephron GFR; SNFF: single-nephron FF; orange lines: single-nephron autoregulation mechanisms; dashed orange lines: some supportive findings in the literature; dashed black line: authors’ hypothesis.

Figure 2 depicts a flowchart of how the parameters of the glomerular hemodynamics of a single nephron should be analyzed after a change in either AA or EA resistance or both without missing links. This flowchart will help eliminate the logical flaws addressed in issue 6.

**FIGURE 3:**
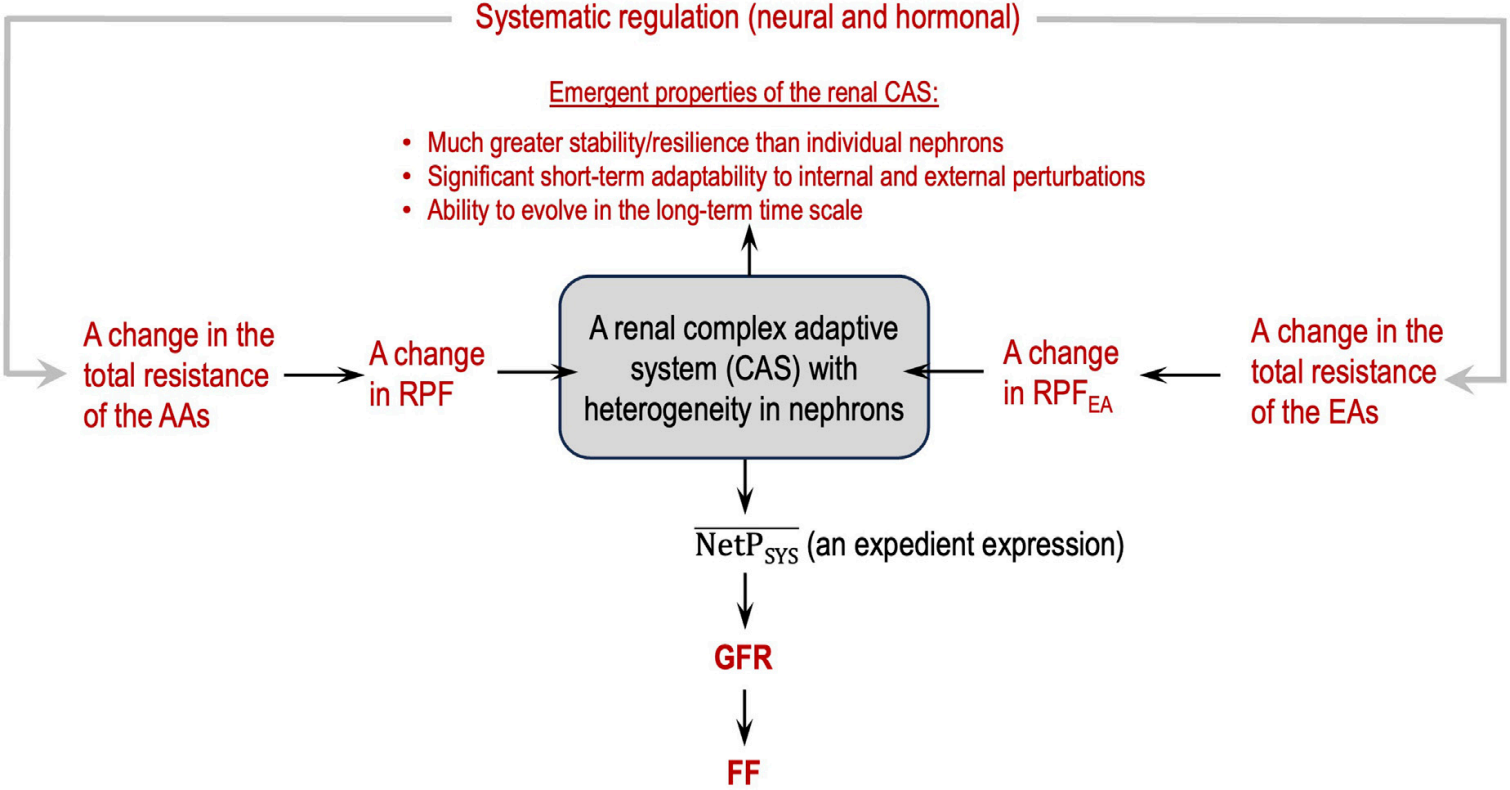
Renal complex adaptive system (CAS) for glomerular filtration with emergent, macroscopic properties (in red).

Figure 3 depicts a flowchart of how to view glomerular hemodynamics at the two-kidney level by considering the two kidneys as a renal CAS.

## 5 A comprehensive analysis to understand the impact of EA constriction on glomerular hemodynamics

Guided by the flowcharts given in Figures 2, 3, issue 6 is resolved in this section by applying Eq. 8 and Hypothesis 3 and Hypothesis 4b.

### 5.1 The essential roles of the EAs and EA baseline resistance

When renal autoregulation maintains stable RPF and GFR, why is the P_GC_ higher than that in the peripheral tissue and relatively stable throughout the glomerular filtration (Figure 1A)? A normal EA tone (a certain level of constant constriction) maintains the P_GC_ high and relatively constant (Savedchuk et al., 2023). This is the essential role of the EAs. This essential role is conditional upon the renal autoregulation of RPF functioning normally.

A normal EA tone results in normal EA resistance (level-0 resistance). This normal EA resistance is the baseline when renal autoregulation mechanisms function normally. EA constriction means that the EAs constrict more than the level of the normal EA tone; thus, EA resistance becomes higher than the baseline. EA dilation means that the EAs constrict less than the normal EA tone; thus, EA resistance becomes smaller than the baseline.

Throughout our analysis, each level of EA constriction sets a new baseline for EA resistance. EA constriction/dilation means that the EAs constrict more/less than a corresponding level of EA constriction.

### 5.2 Comprehensive analysis of the effect of EA constriction on glomerular hemodynamics

The data on RPF, GFR, and FF of the healthy man mentioned above when we addressed Hypothesis 4 are used to facilitate the analysis. Due to renal heterogeneity, glomerular hemodynamics is analyzed at the system level not the SN level.

#### 5.2.1 Slight EA constriction, which sets a level-1 baseline EA resistance

This situation does not reduce RPF significantly or at all but redistributes RPF (∼600) to RPF_EA_ and GFR (Eq. 8). The more the EAs constrict, the more $\bar{{\text{NetP}}_{\text{SYS}}}$ , GFR, and FF increase. According to Hypothesis 3, the EA should stop constricting and dilate (with reference to level 1-EA baseline resistance) to reduce the GFR to normal.

#### 5.2.2 Moderate EA constriction, which sets a level-2 baseline EA resistance, e.g., EA resistance ≈1.5-fold of level-0 EA baseline resistance or RPF_EA_ ≈ 316.7, 2/3 of the original (475)

This situation causes a reduction in RPF and an increase in both $\bar{P_{\text{GC}\_\text{SYS}}}$ and $\bar{\pi_{\text{GC}\_\text{SYS}}}$ , in general. How it influences $\bar{{\text{NetP}}_{\text{SYS}}}$ , GFR, and FF depends on the exact degree of EA constriction and the concrete RPF. If the resulting $\bar{{\text{NetP}}_{\text{SYS}}}$ is around normal, then the GFR is around normal, but FF increases because of a reduction in RPF. If RPF_EA_ remains approximately 316.7, RPF needs to be adjusted to 441.7 (441.7 = 125 + 316.7, Eq. 8) to maintain the GFR at approximately 125.

According to Hypothesis 4b, RPF of approximately 441.7 is the critical point for RPF_EA_ of approximately 316.7. If RPF > or <441.7, then GFR > or < normal, $\bar{{\text{NetP}}_{\text{SYS}}}$ > or < normal, and FF should decrease or increase. According to Hypothesis 3, the EA should dilate or constrict with reference to the level-2 baseline EA resistance. Therefore, level-2 EA constriction is certain to cause a reduction in RPF and an increase in FF but embraces multiple possibilities for the values of $\bar{{\text{NetP}}_{\text{SYS}}}$ and GFR.

#### 5.2.3 Severe EA constriction, which sets a level-3 baseline EA resistance, e.g., EA resistance = or > 3-fold of level-0 EA baseline resistance or RPF_EA_ ≈ 158.3 (1/3 of 475)

This situation causes significant reduction in RPF and $\bar{{\text{NetP}}_{\text{SYS}}}$ , which leads to a decrease in the GFR and an increase in FF. Under such circumstances (most likely, renal autoregulation mechanisms have failed to maintain the normal, stable RPF), the critical point of RPF with regard to RPF_EA_ of approximately 158.3 is 278.3 (278.3 = 125 + 158.3, Eq. 8), if possible. If RPF > or <278.3, then the EA should dilate or constrict with reference to the level-3 baseline EA resistance to adjust the GFR to be not too far from normal. This means, logically, that even though RPF is reduced significantly, as long as the EA can constrict more, it still has the potential to maintain the normal GFR. FF should always increase at level-3 EA constriction. Practically, the EAs may not be able to constrict more, especially if they are smaller in diameter. In addition, it should be noted that if the reduction of RPF is not due to EA constriction, but due to other reasons, the analysis should still revolve around how to maintain the normal GFR by applying Eq. 8, Hypothesis 3 and Hypothesis 4a. 

#### 5.2.4 More severe EA constriction that causes RPF (e.g., 100) < normal GFR (e.g., ∼120)

This situation can occur under some pathological conditions such as acute renal failure, when both the AAs and EAs constrict significantly as part of systemic vascular constriction. Regardless of how severely the EAs constrict, a normal GFR cannot be maintained. Therefore, administering vasodilator(s) to the AA and EA is a preventive action if acute renal failure is likely to occur and life-saving if acute renal failure is already occurring. This is an exception to Hypothesis 3.

#### 5.2.5 Summary

The analyses in this section are guided by the logical relationships shown in Figures 2, 3, Eq. 8, and Hypothesis 3 and Hypothesis 4b. They are much more comprehensive than those in the literature and in standard physiology textbooks, without logical errors, an address the following two important points:

• The impact of EA constriction on glomerular hemodynamics is quite conditional, depending on concrete situations.
• The parameters that should and should not be analyzed must be differentiated in this context in consideration of renal heterogeneity.

## 6 Conclusion

This article presents the following outcomes. First, it makes clear the need for a shift in epistemology to adopt the concept of the CAS and a designer’s view.

Second, some fundamental equations are modified/improved, and one new equation is established in the conventional paradigm. Four new hypotheses are formulated from the perspective of the CAS with guiding significance for future research.

Third, new insights to understand the role of EAs as resistance arterioles are developed, specifically:

• RPF_EA_ (80% RPF) serves as an adequate reserve of the normal GFR. This reserve becomes significant when renal autoregulation fails to maintain normal RPF, and RPF is significantly low. Theoretically, as long as RPF > normal GFR, EA constriction has the potential to adjust EA resistance to maintain the GFR at a normal level. Therefore, the distribution of 80% RPF to EAs, in particular, plays a protective role in the maintenance of the normal GFR. If the fractions of a normal GFR and RPF_EA_ in RPF are reversed, i.e., normal GFR ≈80% RPF and RPF_EA_ ≈ 20% RPF, the EA will not be able to effectively protect the normal GFR in any scenario. This is the second significance of Eq. 8.
• Having an EA aligned in series with an AA and a glomerulus for each nephron is the *necessary condition* to maintain the normal GFR, whereas having 80% RPF entering the EAs as a significant reserve to maintain the normal GFR when renal autoregulation fails to maintain the normal stable RPF is the *sufficient condition* to maintain the normal GFR. Without the sufficient condition, the kidney will lack resilience and adaptability and will be unable to cope with various internal and external perturbations. This analysis of *necessary and sufficient conditions* is borrowed from cybernetics. It theorizes our understanding of renal autoregulation of many of its functions at the philosophical level. If biomedical research studies adopt this perspective, more insights into the biomedical disciplines will emerge.
• It is possible that if the pre-glomerular resistance increases or decreases inappropriately, the EAs have the sensitivity and potential to constrict or dilate to a certain degree to correct the error. This is because the heterogeneity in the diameters of the EAs and the distribution of EAs with different diameters in the renal cortex in contrast to AAs suggest the substantial power and potential of the EAs in the regulation of glomerular hemodynamics in various ways to maintain the normal GFR.

Future research on glomerular hemodynamics should focus on recognizing patterns of renal heterogeneity in response to various perturbations, dynamic interactions among nephrons, and the emergent properties of the renal CAS.

## Data Availability

The original contributions presented in the study are included in the article further inquiries can be directed to the corresponding authors.

## Footnotes

1 ΔP_GC_ = P_GC_ (Figure 1B) – P_GC_ (Figure 1A) = 60–53 = 7 (mmHg) and ∆Q(x_1_) = Q(x_1_) (Figure 1B) – Q(x_1_;) (Figure 1A) = 42–35 = 7 (mmHg), meaning at x_1_, ∆Q(x_1_) = ΔP_GC_, instantaneous NetP(x_1_) (Figure 1B) = instantaneous NetP(x_1_) (Figure 1A).
